# Verruca Vulgaris Eruption Arising in the Setting of a Tyrosine Kinase Inhibitor

**DOI:** 10.7759/cureus.26006

**Published:** 2022-06-16

**Authors:** Ahava Muskat, Shiri Nawrocki, Yana Kost, Daiva Mattis, Bijal Amin, Beth McLellan

**Affiliations:** 1 Dermatology, Albert Einstein College of Medicine, Bronx, USA

**Keywords:** breast cancer, human papillomavirus, verruca vulgaris, wart, tyrosine kinase inhibitor

## Abstract

A 71-year-old female with breast cancer presented with a generalized papular rash that began following the initiation of rebastinib. Examination revealed scattered pink to skin-colored verrucous papules on the forehead, extremities, and back. A biopsy showed hyperkeratosis, hypergranulosis, digitated epidermal hyperplasia, and dilated blood vessels at the tips of dermal papillae consistent with verruca vulgaris. The patient discontinued rebastinib due to muscle weakness and the lesions resolved.

Rebastinib is an antineoplastic agent that targets several tyrosine kinases. Tyrosine kinase inhibitors (TKI) frequently cause cutaneous adverse events, but to date, there have been no reported cases of a verruca vulgaris eruption arising in the setting of TKI treatment.

Recent studies indicate that TKIs can have immunosuppressive effects by decreasing T-cell levels. We postulate that rebastinib induced an immunosuppressive state in our patient which permitted human papillomavirus (HPV) proliferation. To our knowledge, this is the first report describing a verruca vulgaris eruption with TKI therapy.

## Introduction

Rebastinib is a novel antineoplastic agent that targets several tyrosine kinases, including the angiopoietin-1 receptor TIE-2, vascular endothelial growth factor receptor-2 (VEGFR-2), and those in the Src family, which are responsible for angiogenesis, cellular proliferation, and the cellular stress response [1]. It is currently being studied in various clinical trials for the treatment of multiple metastatic solid tumors. Tyrosine kinase inhibitors (TKI) frequently cause cutaneous adverse events. In one study of patients with chronic myeloid leukemia (CML) on imatinib, cutaneous hypopigmentation was seen in almost 50% of patients. It is hypothesized that this effect is due to imatinib’s inhibition of c-Kit, which is normally expressed on melanocytes [2,3]. After hypopigmentation, the most common cutaneous reactions observed were eyelid edema, xerosis, eczematous eruption, and melasma [3]. Second-generation TKIs, nilotinib and dasatinib, exhibit greater specificity for the bcr-abl gene and reduced affinity for c-Kit, and therefore induce fewer side effects than imatinib [2]. However, cutaneous toxicities secondary to nilotinib and dasatinib have been reported in the literature. In one study, pruritus and generalized rash were seen in up to 17% of patients taking nilotinib [2]. In phase II trial of dasatinib, 35% of participants experienced cutaneous side effects [2]. Isolated cases of sweet syndrome, alopecia, panniculitis, and vasculitis have been reported as well [4]. The cutaneous toxicities of TKIs are dose-dependent and typically reversible after discontinuation of the drug [4].

Despite the plethora of literature describing cutaneous toxicities of TKIs, we present a novel case of eruptive verruca vulgaris occurring in the setting of rebastinib initiation and resolving shortly after its discontinuation. To our knowledge, there have been no reported cases of a verruca vulgaris eruption arising in the setting of TKI treatment.

## Case presentation

**Figure 1:**
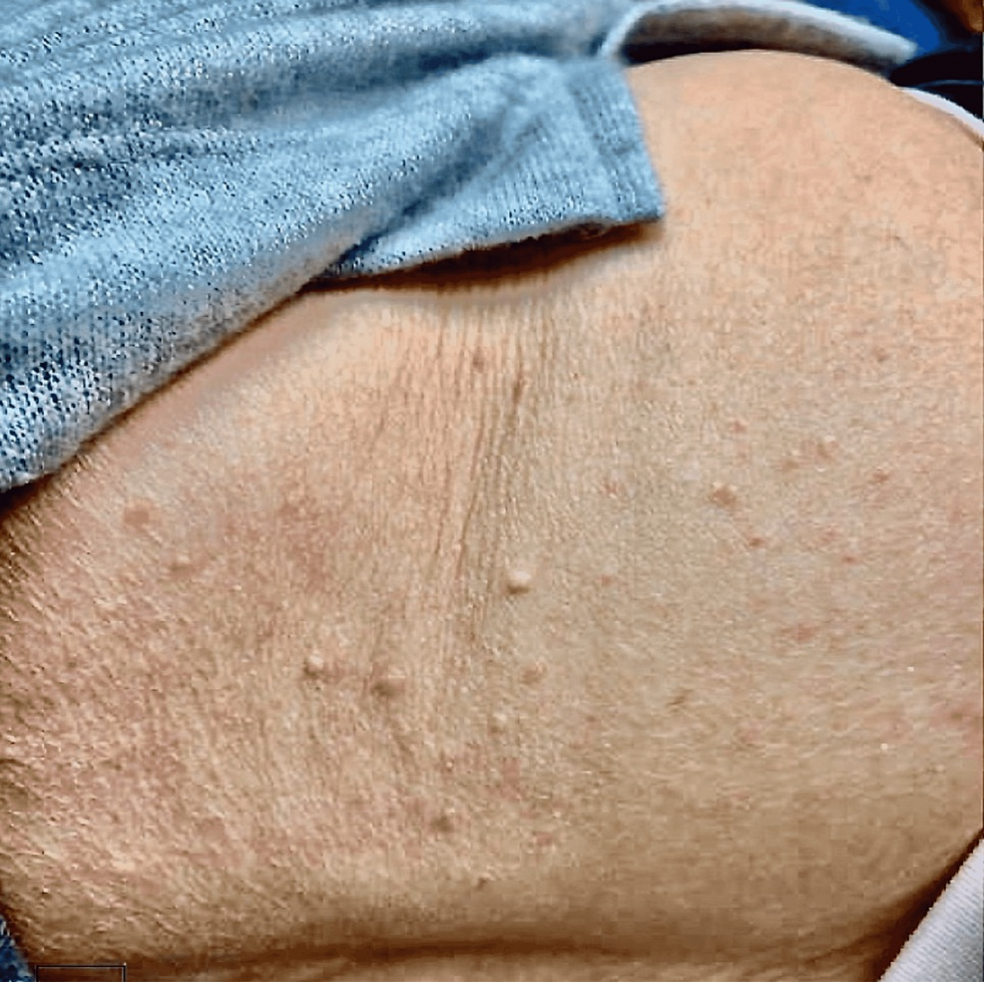
Clinical photograph shows numerous pink and skin-colored papules on the abdomen after starting rebastinib.

A 71-year-old female with stage IV breast cancer presented with a generalized papular eruption that began two weeks following initiation of rebastinib. The patient had no prior history of cutaneous reactions to medications. Physical examination revealed dozens of scattered pink to skin-colored smooth papules on the forehead, extremities, and back (Figure 1).

**Figure 2:**
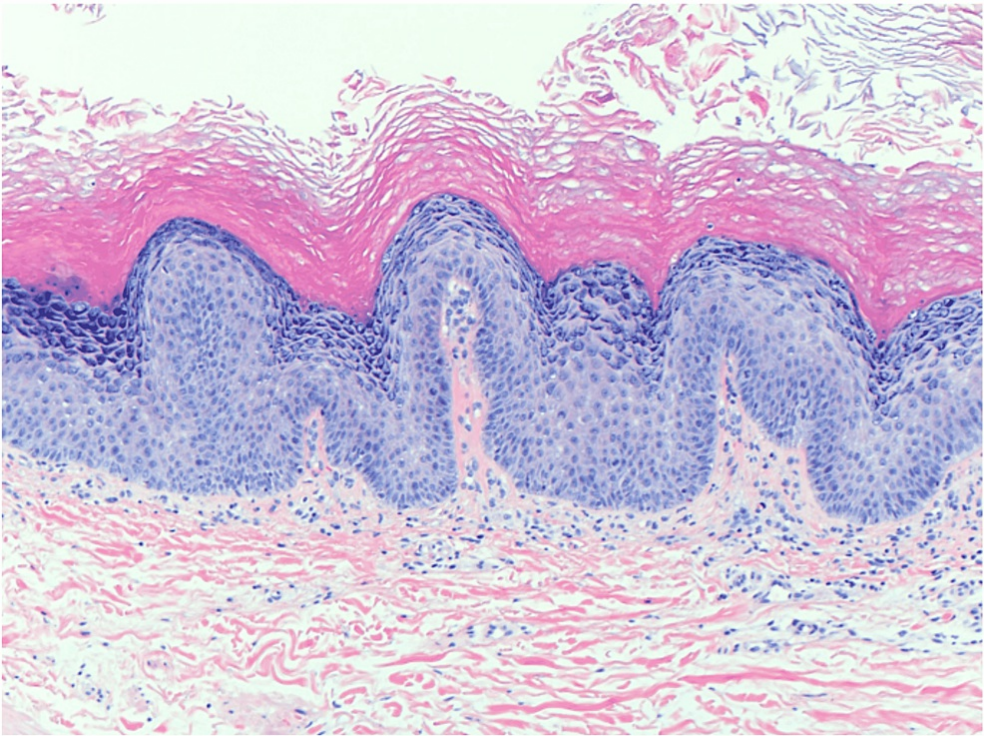
Histopathology of a lesion on the patient’s right lower back after starting rebastinib. The image is showing hyperkeratosis, hypergranulosis, digitated epidermal hyperplasia and dilated blood vessels at the tips of dermal papillae, consistent with verruca vulgaris (hematoxylin and eosin, 200x).

A punch biopsy of a lesion on her right lower back revealed hyperkeratosis, hypergranulosis, digitated epidermal hyperplasia, and dilated blood vessels at the tips of dermal papillae consistent with verruca vulgaris (Figure 2).

**Figure 3:**
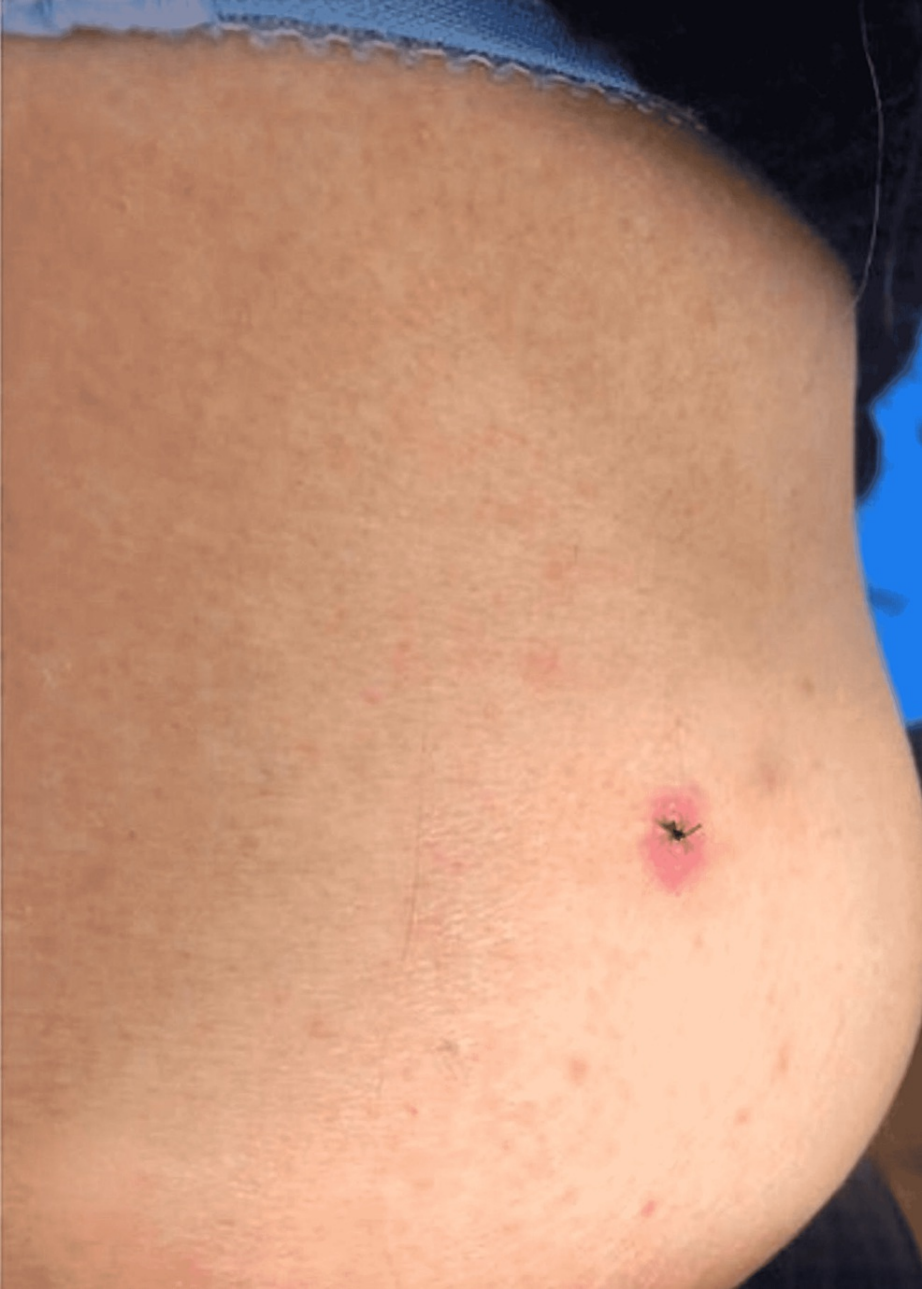
Clinical photograph shows complete resolution of eruption after discontinuation of rebastinib.

Two weeks later, the patient discontinued rebastinib due to muscle weakness. Complete clearance of the lesions was observed at her follow-up appointment, one week after discontinuation of the medication (Figure 3).

## Discussion

Recent studies indicate that TKIs can have both immunomodulatory and immunosuppressive effects. It is thought that TKIs may cause immunosuppression by reducing phosphorylation of Bruton's tyrosine kinase (BTK), and indirectly its substrate phospholipase C gamma 2 (PLCγ2), which causes downstream B-cell impairment [5]. TKIs have also been shown to decrease levels of T-cells in vitro and in vivo. In vitro, decreased expression of cluster of differentiation (CD)69 and CD25, known early T-cell activation markers, were seen in multiple studies [6]. In humans, total T-cell, CD4+, and CD8+ T-cells were decreased in peripheral blood smears of patients taking TKIs. Additional mechanisms involved in T-cell impairment include abnormal T-cell receptor signaling, cytokine production, and proliferation [7]. Furthermore, decreased expression of cytokines interleukin-4, interleukin-10, and transforming growth factor has been reported secondary to the use of the TKIs imatinib and dasatinib [8]. While there are conflicting studies that show that TKI's can potentially stimulate immune activation in some cases, their roles in the setting of T-cell maturation, stimulation, and activation are widely believed to be inhibitory as reproduced in multiple studies [9]. 

Additionally, vascular endothelial growth factor/vascular endothelial growth factor receptor (VEGF/VEGFR) inhibitors, like sorafenib, have been shown to inhibit the stimulation of T-cells in cell culture and murine models, due to the downregulation of costimulatory markers on dendritic cells which are necessary for T-cell maturation [10]. Similarly, one study demonstrated irreversible inhibition of human peripheral T-cells and elevated levels of apoptosis of T-regulatory cells in the setting of sorafenib treatment [11]. As rebastinib inhibits multiple tyrosine kinases and VEGFR-2, we propose that T-cell function was likely impaired in our patient who developed a verruca vulgaris eruption.

Decreased T-cell counts can lead to impaired cell-mediated immunity and consequent human papillomavirus (HPV) infection, as seen in patients with HIV or transplant recipients [12]. Notably, imatinib has been shown to attenuate the immune response to cytomegalovirus and the Epstein-Barr virus in an analogous manner [13]. Similarly, reactivation of the varicella-zoster virus due to reduced cell-mediated immunity has been reported in some patients treated with imatinib [9]. 

We postulate that rebastinib induced an immunosuppressive state in our patient which permitted HPV proliferation and a consequent verruca vulgaris eruption. Resolution with cessation of the drug provides support for this hypothesis.

## Conclusions

We present this case to improve recognition of verruca vulgaris secondary to TKI use and thereby expand the collection of known cutaneous adverse events of TKI therapy. Unlike other certain cutaneous toxicities, verruca vulgaris is a benign eruption that does not require cancer treatment discontinuation. We hope that our case presentation will improve knowledge of and the recognition of this entity to limit possible cancer treatment interruption.
